# Characteristics and antiviral treatment eligibility of patients diagnosed with hepatitis B at a teaching hospital in Ghana: Implications for prevention and management

**DOI:** 10.1371/journal.pone.0302086

**Published:** 2024-08-22

**Authors:** Joseph Daniels, Yvonne A. Nartey, Francis Djankpa, Jacques Simpore, Dorcas Obiri-Yeboah

**Affiliations:** 1 National Centre for Radiotherapy, Oncology and Nuclear Medicine, Korle Bu Teaching Hospital, Accra, Ghana; 2 Department of Microbiology and Immunology, School of Medical Sciences, University of Cape Coast, Cape Coast, Ghana; 3 Department of Internal Medicine and Therapeutics, School of Medical Sciences, University of Cape Coast, Cape Coast, Ghana; 4 Department of Physiology, School of Medical Sciences, University of Cape Coast, Cape Coast, Ghana; 5 Centre de Recherche Biomoléculaire Pietro Annigoni (CERBA), Ouagadougou, Burkina Faso; Kwame Nkrumah University of Science and Technology, GHANA

## Abstract

Hepatitis B virus (HBV) infection poses a considerable public health challenge in limited-resource settings especially in the sub-Saharan African region. Even though HBV infection is incurable, timely treatment is effective in preventing disease progression to liver cirrhosis or hepatocellular carcinoma. However, not all infected patients require treatment. The aim of the study was to determine the clinical, immunological, and virological profiles of treatment naïve patients with HBV infection, seen at the outpatient clinic of the Cape Coast Teaching Hospital. Additionally, the study sought to determine the antiviral treatment eligibility rate based on the 2015 guidelines of the World Health Organization (WHO) compared with the new 2024 guidelines. A hospital-based cross-sectional study involving total sampling of 220 treatment naïve HBV surface antigen positive clients was carried out. A structured questionnaire was used to collect data that were analyzed with STATA version 16. The median age at diagnosis was 34 years (IQR 26.0–41.5) with a male to female ratio of 1:1.5. A total of 138 participants (62.7%) were diagnosed with HBV infection following voluntary testing. There was a median delay of 8.5 months (IQR 3.0–22.5) between initial diagnosis and patients’ presentation for medical care. In all, 24 patients (10.9%) had abnormal clinical examination findings, 172 patients (78.2%) had HBV DNA levels ≤ 2000 IU/ml whereas 8 (3.6%) were seropositive for the HBV envelope antigen. A few patients had concomitant human immunodeficiency virus (2.7%) and hepatitis C virus (1.4%) infections. Treatment eligibility rate based on the WHO 2015 guidelines was 6.4% (n = 14), however, with the updated 2024 guidelines, treatment eligibility was 42.3% (n = 93). Increasing the screening rate among the general population, early linkage to clinical care of screen positives and vaccination of screen negatives will help reduce HBV-related clinical conditions in resource-limited settings.

## Introduction

Estimated global prevalence rates in 2022, based on data from 170 countries suggest that about 254 million individuals worldwide have chronic hepatitis B virus (HBV) infection [1]. The overall global lifetime risk of HBsAg seropositivity is over 60%, comparable to that of high-endemic regions [2]. In contrast, intermediate-endemic regions have an estimated lifetime risk of 20–60%, while low-endemic regions have a lifetime risk of less than 20% [3]. The estimated number of new viral hepatitis infections decreased from 2.5 million in 2019 to 2.2 million in 2022, of which 1.2 million were new HBV infections. This reduction is attributed to the effectiveness of prevention measures, such as hepatitis B vaccination [3]. Hepatitis B virus infects the liver and is known to be a major global cause of both acute and chronic hepatic diseases [4]. Hepatitis B infection is estimated to cause between 500,000 and 1.2 million deaths worldwide annually through chronic hepatitis and associated complications such as liver cirrhosis and hepatocellular carcinoma (HCC) [5,6]. Hepatitis B accounts for 80% of all liver cancer cases and is the third most common cause of cancer deaths worldwide [7]. Hepatitis B-related deaths are estimated to have risen from 820,000 in 2019 to 1.1 million in 2022 due to several factors, including the ageing population cohort with hepatitis B infection and COVID-19-related disruptions that hindered the expansion of treatment access in many low- and middle-income countries [6].

The highest prevalence of HBsAg seropositivity is found in the WHO Western Pacific Region and Africa with 97 million and 65 million people respectively reported to be chronically infected [8]. In Ghana, there is high (>8%) prevalence of hepatitis B infection per the World Health Organization (WHO) classification [9]. The national prevalence of HBV infection in Ghana is estimated to be about 12.3% [10]. Hepatitis B virus infection requires great attention in Ghana due to its significant public health importance.

Not all patients who test positive for HBsAg require treatment. Patients’ eligibility for the commencement of antiviral therapy is dependent on key factors such as age, HBV viral load, serum alanine transaminase (ALT) levels, aspartate to platelet ratio index (APRI), clinical evidence of liver cirrhosis, comorbidities such as diabetes mellitus, and concomitant hepatitis C virus (HCV), hepatitis D virus (HDV) or human immunodeficiency virus (HIV) coinfections [11–13]. It is important to accurately classify patients based on these parameters, since this directly influences clinical decisions regarding antiviral therapy. These parameters are also important in identifying patients at risk of progression to liver cirrhosis or cancer, and as such determine which individuals require immediate initiation of antiviral therapy.

In many countries, a significant proportion of HBV-infected individuals remain undiagnosed. Even when a diagnosis is made, the number of people receiving treatment remains very low [13]. With the high endemicity of chronic HBV infection in Ghana, it is important to determine the proportion of HBV patients who require treatment. It is equally important to identify those who present for the first time with complications such as cirrhosis, hepatic decompensation and HCC, since this highlights missed opportunities for the care and management of persons living with HBV infection.

This study aimed to describe the clinical, immunological, and virological profiles of treatment naïve patients with HBV infection who were presenting for clinical care for the first time at the viral hepatitis/ sexually transmitted infections (STIs) outpatient clinic at the Cape Coast Teaching Hospital in Ghana. Additionally, the study sought to determine the antiviral treatment eligibility rate based on the 2015 guidelines of the WHO compared with the new 2024 guidelines.

## Methods

### Study design and setting

This research was a hospital-based quantitative cross-sectional study. Participants were recruited into the study from viral hepatitis/ STIs clinic at the Cape Coast Teaching Hospital (CCTH) which is situated at the northern part of the Cape Coast metropolis in the Central Region of Ghana. This hospital is a 400-bed capacity facility that serves as the tertiary care referral center for both private and public healthcare institutions in the central and western regions of Ghana. The clinic started operating in 2009 and had registered about 3000 clients with HBV infection by 2021. The hospital provides comprehensive HBV care and runs a specialist-led gastrointestinal (GI) and hepatology clinic that manages patients with diseases of the GI and hepatobiliary tracts including those with complications related to viral hepatitis such as liver cirrhosis and hepatocellular carcinoma.

### Study participants

Due to interruptions caused by the COVID-19 pandemic, participants were recruited intermittently over a cumulative period of 11 months, encompassing periods from December 10, 2019, to June 09, 2020, and November 03, 2020, to February 09, 2021. Samples were collected at the clinic once a week. There was total population sampling of all adult clients diagnosed with HBV infection who were seeking clinical care at CCTH during the sampling period. Patients included in this study were treatment naïve adult clients who were 18 years or older, with a positive HBsAg test who provided written informed consent. All patients with known HBV/HIV coinfection who were already on highly active antiretroviral therapy (HAART) were excluded from the study as well as individuals on antiviral therapy for other conditions if their medications were known to be simultaneously active against HBV infection. All patients who had been previously treated for HBV infection were also excluded from the study.

### Study size

All clients attending the clinic who met the inclusion criteria (n = 231 over the recruitment period) were offered the opportunity to be part of the study. However, 11 (4.8%) eligible clients declined to provide informed consent to participate in the study for personal reasons. In all, 220 patients were recruited into the study.

### Ethical considerations

The study was approved by the institutional review board of CCTH (Reference number: CCTHERC/EC/2019/084). All patients’ information including laboratory test results were kept private and confidential. The data of the participants were anonymized prior to analysis. Throughout this study, there was strict adherence to the prescribed standards of acceptable scientific and ethical behavior. All relevant COVID-19 protocols were observed during interactions with patients and data collection. All the study participants provided written informed consent prior to their recruitment into the study.

### Data sources

Data regarding socio-demographic characteristics, past medical and family history as well as other relevant factors linked to HBV infection were collected through a structured questionnaire which comprised 32 closed-ended questions. Relevant data were also abstracted from patients’ hospital records with their consent. The questionnaire was pretested in a similar out-patient setting to determine its validity and reliability to ascertain its appropriateness for this study. Twenty-two (22) clients were selected for pretesting, representing 10% of the actual sample size. Tau-equivalent reliability, also known as Cronbach’s Alpha (α) was computed to determine the reliability coefficient. The Alpha value obtained was 0.72 (number of items = 32), therefore this research instrument was deemed reliable and acceptable for collecting useful data for the study.

### Laboratory procedures/analysis

Five milliliters (5mls) of venous blood sample were drawn from the antecubital region of each patient under sterile conditions into a separate purple-capped ethylenediamine tetra acetic acid vacutainer tube and labelled appropriately. The samples were immediately transported on ice from CCTH to the physiology laboratory of the School of Medical Sciences, University of Cape Coast and analyzed. Single-use commercially available First Response^®^ HBsAg rapid test kits (Premier Medical Corporation Private Limited, India) were used to qualitatively detect HBsAg in the plasma specimen of patients. Using the COBAS^®^ AmpliPrep / COBAS^®^ TaqMan^®^ HBV Test, version 2.0 (Roche, Switzerland) (CAP-CTM) and in accordance with the manufacturer’s instructions, the HBV viral load was measured from 400μl of patients’ blood [14]. The Biopanda HBV Combo Rapid Test (RAPG-HBV-001) (Biopanda, United Kingdom) was utilized to qualitatively detect HBsAg, HBsAb, HBeAg, HBeAb, and HBcAb in patients’ blood samples. The ChemWell^®^ 2910 Automated EIA and Chemistry Analyzer (Awareness Technologies, USA) was used to measure serum electrolytes, blood urea nitrogen (BUN) and liver enzymes including aspartate and alanine transaminases, strictly following the manufacturer’s protocol. Complete blood counts (CBCs) were determined using the Mindray BC-2800 Auto Hematology Analyzer (China) [15]. All the patients were also tested for HIV infection using the First Response^®^ HIV 1–2.0 test (Premier Medical Corporation Private Limited, India), OraQuick^**®**^ HIV test (OraSure Technologies, USA) and SD Bioline^™^ HIV-1/2 antibody test kits (Abbott, USA) based on the guidelines for HIV screening and diagnosis among adults, pregnant women and adolescents in healthcare settings [16]. All patients (N = 220) were initially tested with the First Response^®^ HIV 1–2.0 test. Those with reactive results (n = 6) underwent confirmatory tests with the OraQuick^®^ HIV test. All 6 patients were reactive on both tests and hence diagnosed as being HIV-positive. Single use commercially available HCV rapid test kits (Advanced Diagnostic Kit for immunoglobulin to HCV, China) were used to qualitatively detect the presence of anti-HCV immunoglobulins in patients’ samples with sensitivity of 99.0% and specificity of 99.8% [17]. The determination of APRI values was based on hematological and biochemical tests conducted on patients’ samples using the formula: (AST/40/ platelet count (10^9^/L) x 100) [18].

### Statistical analysis

Data were anonymized, coded, statistically cleaned, and analyzed using STATA statistical software package version 16 for Microsoft windows (College Station, TX: Stata Corp LLC). A combination of descriptive and inferential statistics was performed. Socio-demographic and other characteristics of participants were presented as frequencies with percentages. Histogram and Shapiro-Wilk’s test were used to assess normality of the data. Means and standard deviations were used for normally distributed data while median and interquartile range were used for skewed data. For this study, *p* ≤ 0.05 was considered statistically significant at a 95% confidence interval.

## Results

### Socio-demographic and behavioral characteristics

**Fig 1:**
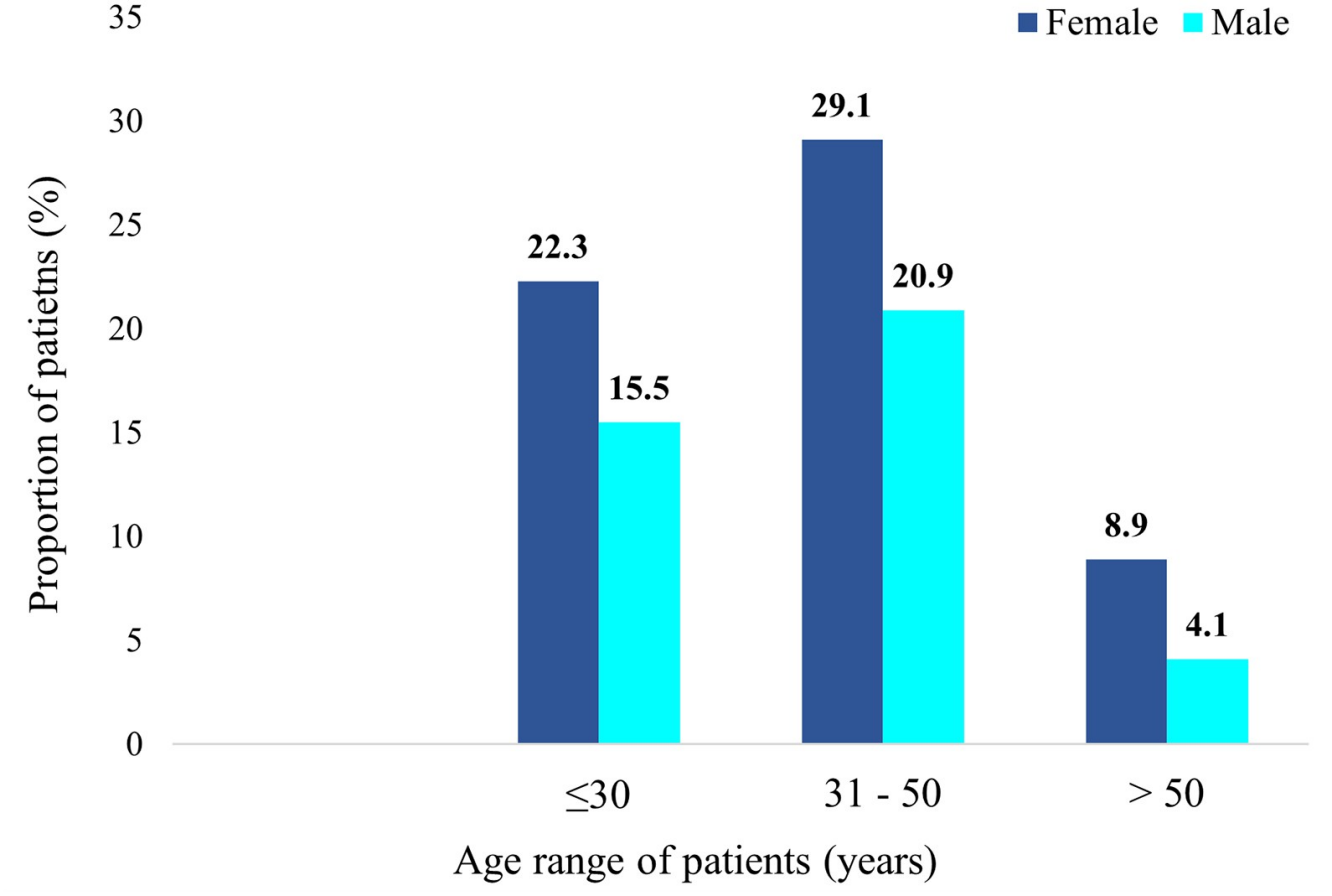
Percentage distribution of patients by age and gender.

In total, 220 individuals with confirmed hepatitis B viral infection participated in this study. A total of 131 (59.6%) of the participants were female whereas 109 (40.4%) were male. There were more females than males in all age categories (Fig 1).

The median age was 34 years (IQR 26.0–41.5). One hundred and eight (49.0%) were single whilst the remaining (n = 112, 51.0%) were either married, divorced, or widowed. Majority (n = 143, 65.0%) were sexually active. The median interval between initial diagnosis and presentation at the clinic was 8.5 months (IQR 3.0–22.5), with about a third of the patients (61.8%) attending their first visit to the clinic more than a year after initial diagnosis (Table 1).

**Table 1:** Socio-demographic and behavioral characteristics (n = 220).

Variables	Categories	Number (n)/ Median	Percentage (%)/ IQR
**Age (years)**	Median	34	26.0–41.5
	≤ 30	83	37.7
	31–50	110	50.0
	> 50	27	12.3
**Marital status**	Single	108	49.0
	Married	100	45.5
	Divorced/ Widowed	12	5.5
**Sexual activity**	Active	143	65.0
	Inactive	77	35.0
**Pregnancy status of females (n = 131)**	Pregnant	9	6.9
	Not-pregnant	122	93.1
**Time since initial diagnosis (years)**	Median	8.5 months	3.0–22.5
	< 1	136	61.8
	1–5	65	29.6
	> 5	19	8.6
**History of alcohol intake**	Yes	30	13.6
	No	190	86.4
**Smoking habit**	Smokers	6	2.7
	Non-smokers	214	97.3

IQR: Interquartile range.

**Fig 2:**
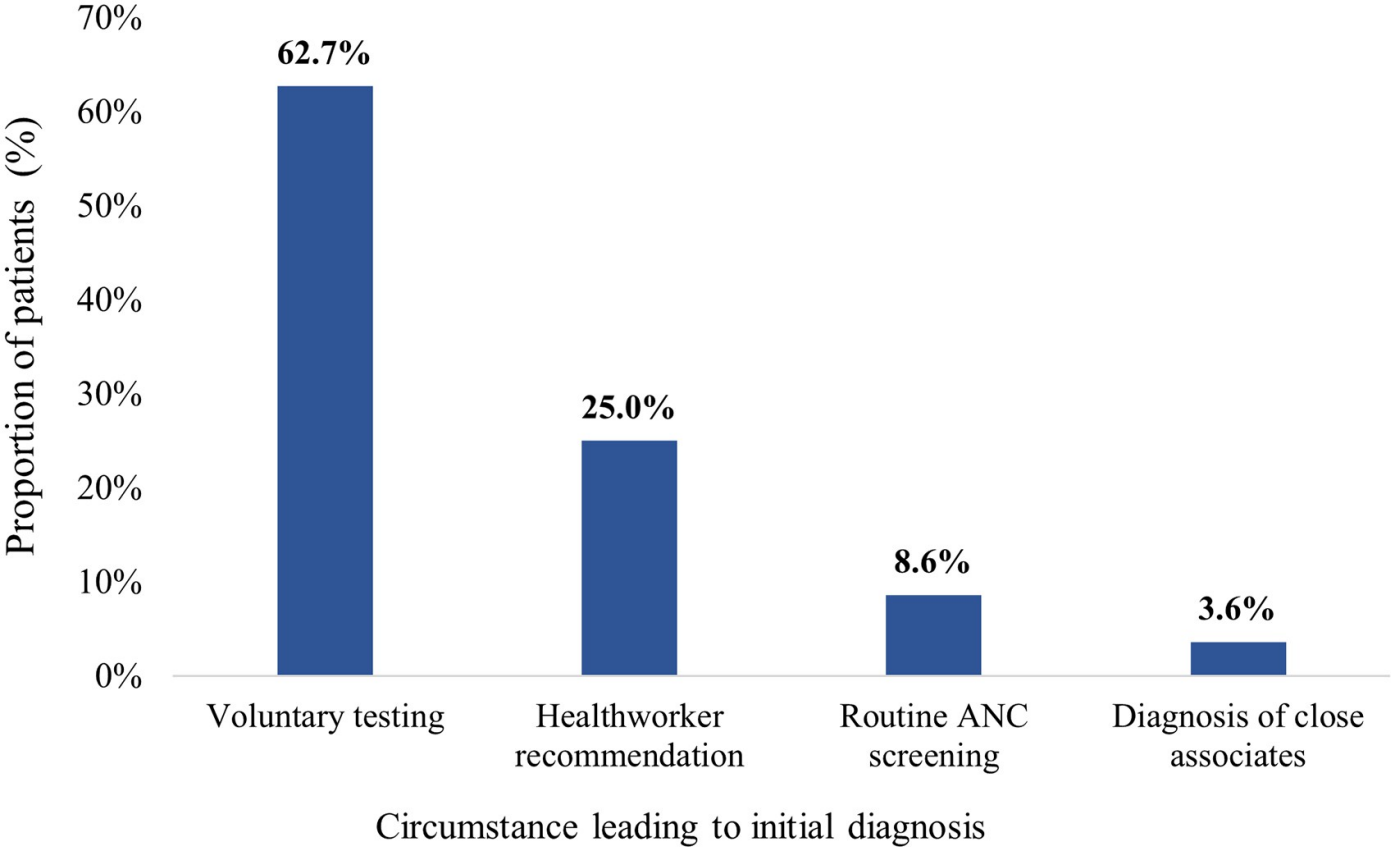
Circumstances leading to initial diagnosis among study participants (N = 220). ANC: Antenatal care.

Most of the participants (n = 138, 62.7%) were initially diagnosed with HBV infection due to voluntary testing whereas 19 females (8.6%) were diagnosed as part of routine antenatal care (ANC) screening. A few (n = 8, 3.6%) were diagnosed via screening after the diagnosis of close associates (sexual partners & family members) whereas the remainder (n = 55, 25.0%) were diagnosed after testing pursuant to the recommendation of a health-worker (Fig 2).

**Fig 3:**
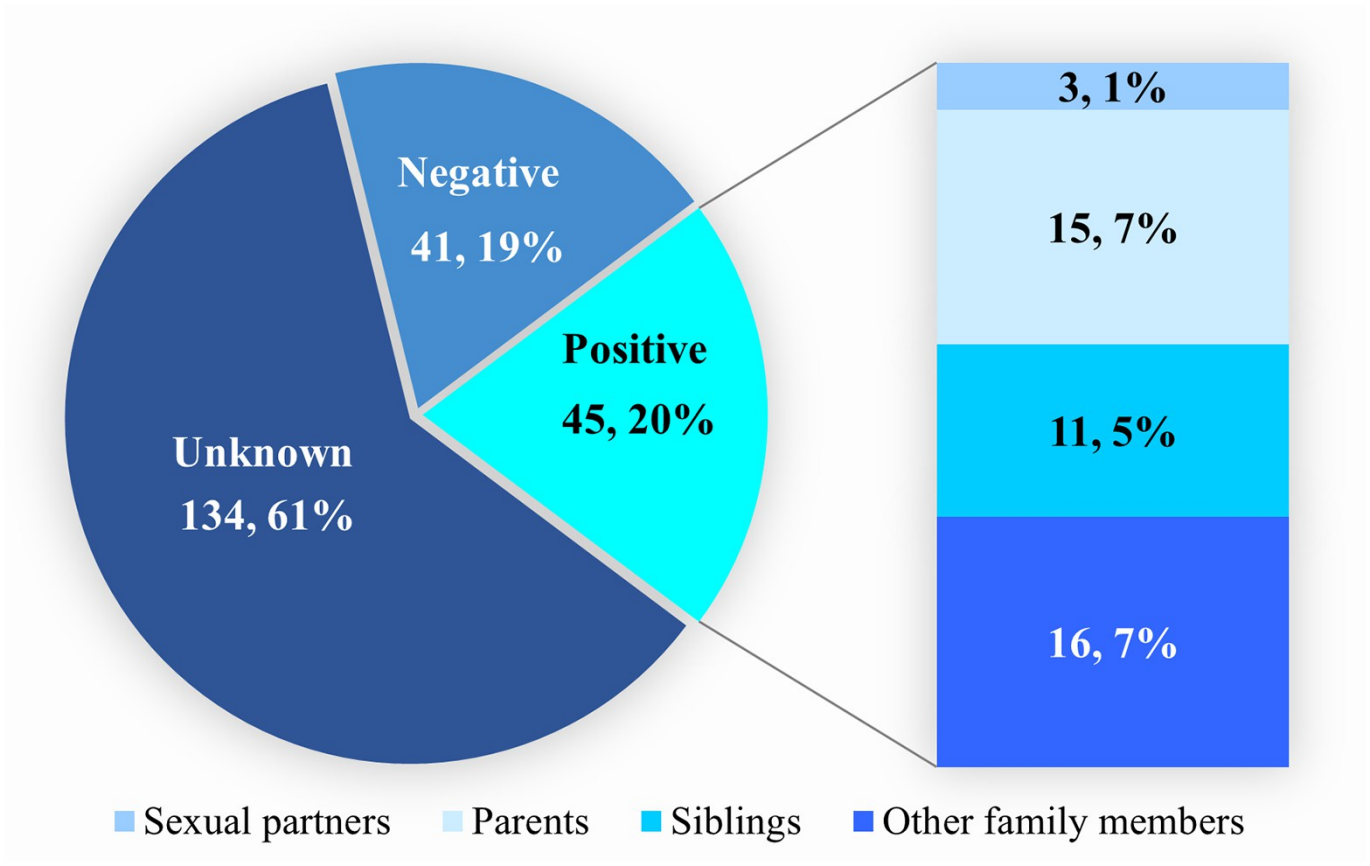
Distribution of HBV seropositive family members and sexual partners of the study participants (N = 220).

Approximately 60.9% did not know the HBsAg status of their immediate family members whereas 20.5% had a positive family history of HBV infection. In all, 15 (6.8%) had parents with HBV infection as illustrated in Fig 3.

**Fig 4:**
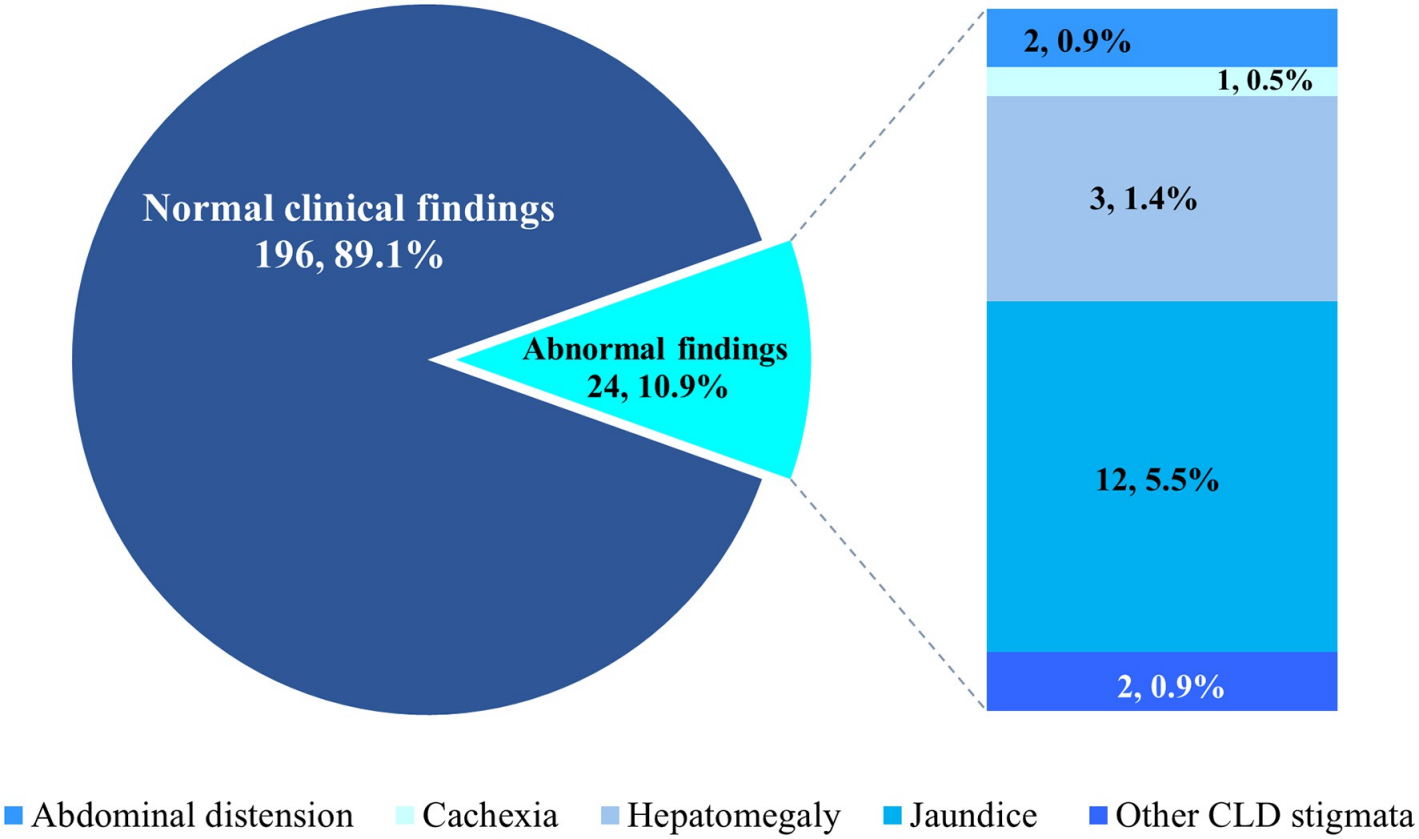
Distribution of clinical examination findings among patients (N = 220). CLD: Chronic liver disease.

In all, 24 patients (10.9%) had abnormal clinical examination findings. There were 12 patients (5.5%) who had icterus (jaundice), whereas 3 (1.4%) had hepatomegaly as illustrated in Fig 4 which shows the distribution of clinical examination findings among patients. Two patients (0.9%) each had abdominal distension and other stigmata of chronic liver disease.

### Laboratory characteristics of patients

Most of the participants had aspartate transferase (AST) and alanine transaminase (ALT) levels within the normal range, 131(59.6%) and 195(88.6%) respectively. One hundred and eighty participants (81.8%) also had alkaline phosphatase (ALP) levels within the normal range as summarized in Table 2.

**Table 2:** Liver function tests of study participants (N = 220).

Variables	Categories	Number	Percentage (%)
**AST (U/L)**	Normal (0–31)	131	59.6
	Elevated (> 31)	89	40.4
**ALT (U/L)**	Low (< 13)	7	3.2
	Normal (13–69)	195	88.6
	Elevated (> 69)	18	8.2
**ALP (U/L)**	Low (< 50)	15	6.8
	Normal (50–250)	180	81.8
	Elevated (> 250)	25	11.4
**Direct bilirubin (μmol/l)**	Low (< 2)	28	12.7
	Normal (2–11)	163	74.5
	Elevated (> 11)	29	13.2
**Albumin (g/dl)**	Low (< 35)	33	15.0
	Normal (35–52)	178	80.9
	Elevated (> 52)	9	4.1

AST: Aspartate transferase, ALT: Alanine transaminase, ALP: Alkaline phosphatase.

In all, six patients (2.7%) were reactive upon testing with both First Response^®^ HIV 1–2.0 and OraQuick^®^ HIV test kits and hence diagnosed as being HIV-positive. A small proportion of patients (1.4%) had a concomitant HCV infection. Most of the patients (n = 186, 84.6%) had APRI scores ≤ 0.5. Also, 172 patients (78.2%) had HBV DNA levels ≤ 2000 IU/ml. Only one patient (0.5%) had an HBV DNA titer > 20,000 IU/ml (Table 3).

**Table 3:** Other laboratory parameters of study participants.

Clinical parameters	Variables	Number (n = 220)	Percentage (%)
**HIV Status**	Positive	6	2.7
	Negative	214	97.3
**HCV Status**	Positive	3	1.4
	Negative	217	98.6
**APRI score**	≤ 0.5	186	84.6
	> 0.5–1	28	12.7
	> 1.0	6	2.7
**HBV DNA** **(IU/ml)**	< 2000	172	78.2
	2000–20,000	47	21.3
	> 20,000	1	0.5

HIV: Human immunodeficiency virus, HCV: Hepatitis C virus, APRI: Aspartate-platelet ratio index, HBV: Hepatitis B virus, DNA: Deoxyribonucleic acid.

**Fig 5:**
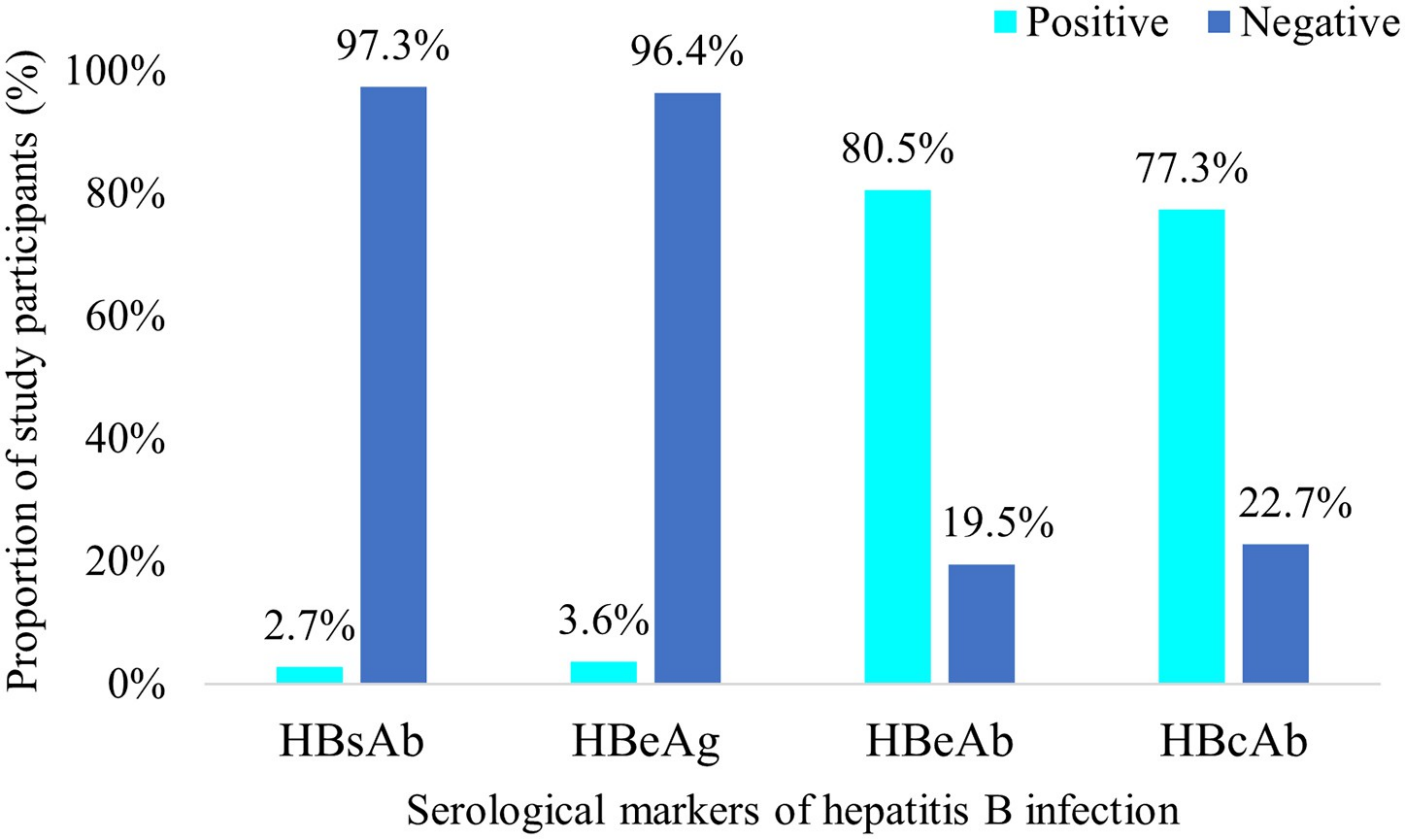
HBV serological profile of patients (N = 220). HBsAb: Hepatitis B surface antibody, HBeAg: Hepatitis B envelope antigen, hepatitis B envelope antibody, hepatitis B core antibody.

The proportion of patients who tested positive for HBsAb and HBcAb were 6 (2.7%) and 170 (77.3%) respectively. Also, 8 patients (3.6%) were seropositive for HBeAg whereas 177 (80.5%) showed seropositivity for HBeAb (Fig 5).

### Treatment eligibility

In all, 14 participants (6.4%) were eligible for immediate initiation of antiviral therapy based on the 2015 WHO guidelines [19] whereas 93 (42.3%) met the expanded treatment-eligibility criteria of the 2024 WHO guidelines and would have qualified for antiviral medication if the updated guidelines had been published at the time of the study [13,20]. The group of patients who met the 2015 WHO criteria for antiviral treatment comprised 8 (57.1%) males and 6 (42.9%) females (Table 4). All patients with e-antigen positive chronic hepatitis and/or APRI > 2 (HBV-related cirrhosis or advanced fibrosis) required treatment as well as individuals with HCV/HIV coinfection and HCC. Among HBeAg-positive patients, treatment was required if ALT > 2x ULN or viral load ≥20,000IU/ml. For HBeAg-negative patients, indications for treatment were ALT ≥ 2x ULN or viral load ≥2,000IU/ml. None of the pregnant females in the study was found to be eligible for either immediate commencement of long-term antiviral treatment or prophylaxis for mother-to-child HBV transmission per the 2015 WHO criteria.

**Table 4:** Characteristics of patients who were eligible for antiviral therapy.

Antiviral treatment eligibility	Based on 2015 WHO Guidelines		Based on 2024 WHO Guidelines	
	Frequency n/N	Percentage (%)	Frequency n/N	Percentage (%)
Not eligible for treatment	206/220	93.6	127/220	57.8
Treatment eligible	14/220	6.4	93/220	42.3
Sex				
Male	8/14	57.1	38/93	40.9
Female	6/14	42.9	55/ 93	59.1
HBeAg status				
Positive	8/14	57.1	48/93	8.6
Negative	6/14	42.9	85/93	91.4
Serum ALT (U/L)				
≤ 2x ULN (2015) or ≤ ULN (2024)	10/14	71.4	32/93	34.4
> 2x ULN (2015) or > ULN (2024)	4/14	28.6	61/93	65.6
Advanced liver dx				
APRI > 2	1/14	7.1	1/93	1.1
APRI > 1	2/14	14.3	6/93	6.5
APRI > 0.5	3/14	21.4	34/93	36.6
Pregnant women (n = 9)				
Requiring treatment	0/9	0	5/9	55.6
Co-infection status				
HIV-HBV	6/14	42.9	6/93	6.5
HCV-HBV	3/14	21.4	3/93	3.2
Comorbidities	2/14	14.3	24/93	25.8

HBeAg: Hepatitis B envelope antigen, ALT: Alanine transaminase, ULN: Upper limit of normal range, dx: Disease APRI: Aspartate-platelet ratio index, USG: Ultrasonography, HCC: Hepatocellular carcinoma, HIV: Human immunodeficiency virus, HBV: Hepatitis B virus, HCV: Hepatitis C virus.

Based on the updated WHO 2024 criteria [20], 38 (40.9%) males and 55 (59.1%) females would have qualified for antiviral medication if the guidelines were already published at the time of the study (Table 4). Five (55.6%) out of the nine pregnant women in the study were eligible for long-term antiviral treatment per the updated guidelines. All patients older than 12 years were deemed eligible for treatment if they had significant fibrosis or cirrhosis regardless of HBV DNA or ALT levels (based on clinical criteria for cirrhosis or APRI >0.5 in adults or transient elastography >7kPa). Additionally, patients with HBV DNA >2000 IU/mL and ALT level > ULN qualified to be treated.

## Discussion

This study aimed to describe the clinical, immunological, and virological profiles of treatment naïve patients with HBV infection who were presenting for clinical care for the first time at the viral hepatitis/ STIs outpatient clinic at CCTH in Ghana, and to furthermore determine the treatment eligibility of these patients. The major findings were a median age of presentation in the 4^th^ decade of life, an average 8.5-month lag period between first diagnosis and first clinic visit. The majority of patients were only identified as HBsAg-positive following voluntary testing rather than any health service-led initiative. Furthermore, our findings identified that approximately 36% more patients (almost 7 times more) were eligible for antiviral medication when comparing the WHO 2024 to 2015 treatment guidelines.

The median age of the participants in this study of 34 years is comparable to the results of previous studies that were also focused on adult HBV patients ≥ 18 years [21]. In a meta-analysis of 30 peer reviewed publications with a population pool of over 100,000 Ghanaians, the authors reported the highest prevalence of HBV infection amongst patients aged between 16 and 39 years [10]. In sub-Saharan Africa, HCC has been reported to develop at a mean age of 33 years [22]. Individuals aged between 31 and 40 years have been linked with a five-fold increase in the risk of HBV infection relative to other age groups (P = 0.009). The age range reported in this study represents both the reproductive and most productive age groups in the Ghanaian society and may imply loss of productivity due to absence from work for hospital visits and other economic activities among these patients.

There were 42 more females than males in our study population, representing 19.1% of the total number of study participants. Two previous studies also reported a trend of higher numbers of females compared to males in their studies involving patients with hepatitis B infection [23,24]. This female preponderance is partly attributable to the disparity in the general health seeking behavior among both sexes, which is consistent with a study that found that, in Ghana, more women use formal healthcare services compared to men [25]. Women are more at risk of contracting STIs than men [26], which translates into a higher likelihood of disease transmission from men to women than vice versa [26]. Another study also confirmed the existence of gender disparity in the prevalence of infections caused by HBV [27].

In our study, we found that 63.3% of the patients did not have any knowledge about the HBsAg status of their sexual partners. This finding is not in keeping with the WHO recommendation of routine screening of partners of all patients diagnosed with HBV [28]. In all, only 39.1% (n = 85) of the participants were aware of their family’s HBsAg status. Half (n = 45, 52.9%) had HBsAg-seropositive family members. This suggests intrafamilial HBV transmission may be significant in Ghana, warranting further research. Disclosing a positive HBV status enables the individual to receive social support [29] and encourages close contacts to follow preventive measures, such as avoiding shared personal items. However, further research is needed to assess how HBV status disclosure affects vaccination rates in Ghana. Disclosure also prompts HBsAg testing, helping to identify other cases in the family and can encourage seropositive family members to seek treatment, potentially reducing the risk of cirrhosis and liver cancer. A 2020 study in Ghana found that patients were more likely to disclose their status under certain conditions, such as a desire for testing and vaccination of close contacts, the need for social and/or financial support [30]. Chronic HBV, being incurable, contributes to societal stigma. Stigmatization leads some individuals to hide their HBV status, thus preventing them from receiving appropriate medical care [31].

Most of the participants were initially diagnosed via voluntary testing (62.7%) followed by health worker recommendation (25.0%). Similar findings were reported in a previous study that demonstrated that individuals with HBV infection were diagnosed because of self or physician-initiated testing, testing during screening programs and ANC testing [24,32]. The findings of this study indicate that voluntary testing for HBsAg among the adult population is very important in HBV infection case-detection. Community based or opportunistic screening undoubtedly remains an effective tool in picking up positive cases in the general population.

The median time interval between patient’s initial diagnosis and presentation at the specialist clinic was 8.5 months (IQR 3.0–22.5). The majority (61.8%) were seen < 1 year whereas 8.6% were seen > 5 years after initial diagnosis. The reasons for this delay in presentation at the specialist clinic may be associated with both patient and primary care provider factors. In several African countries including Ghana, many patients who test positive for HBsAg during opportunistic screening are unable to afford the additional tests or the cost of therapy because it must be paid for out-of-pocket [10,33,34]. As a result, a lot of patients who test positive for HBV do not proceed immediately to seek medical care or do not complete the required initial evaluation to determine what needs to be done for them. In Ghana, anecdotal evidence suggests that some patients resort to traditional and/or herbal remedies for the initial management of STIs such as HIV, HCV, and HBV as well as other health conditions including cancer, diabetes, hypertensive heart disease and bronchial asthma [35–37]. As a result of this exploration and experimentation with unorthodox medicine it is possible that some patients may present late to the hospital only after all other avenues have failed or when there is obvious disease progression.

According to the WHO, as of 2019, 10.5% of all the people estimated to be HBsAg positive already knew their positive status whereas only 22% of those diagnosed were receiving medical care [4]. This points to the fact that a huge proportion of HBV infected patients do not receive medical care after their diagnosis. Additionally, certain impediments to HBV treatment and care have been reported including barriers relating to sociocultural beliefs and health systems. These sociocultural beliefs include the perception that HBV is a spiritual disease and a form of punishment from God. On the individual level, some patients take HBV infection for granted owing to the absence of specific symptoms of Chronic HBV infection [30]. Furthermore, the financial burden of hospital-centered treatment for HBV infection is considerably high and even prohibitive for some patients [34]. In Ghana, unlike patients with HIV or HBV/HIV coinfection, HBV mono-infected patients must pay for their medications out of pocket in addition to the cost of their routine investigations such as liver function tests, HBV immunological profile and hepatitis B viral load. The inclusion of medications, laboratory analysis and other investigations under the National Health Insurance Scheme could potentially remove this barrier at least partially if not completely. Similar to findings in other countries such as Burkina Faso and Australia, the delay in seeking care for HBV infection could also be because of stigmatization (or fear of it thereof), lack of knowledge and misconceptions about HBV treatment as well as long queues and waiting times at treatment facilities [38,39].

Treatment for viral hepatitis in Ghana is based on the National Guidelines for Prevention, Care and Treatment of viral hepatitis which is in line with the treatment recommendations of the World Health Organization [19]. Approved antiviral agents for treatment of HBV infection in Ghana include Tenofovir disoproxil fumarate, Entecavir, Pegylated interferon and Emtricitabine among several others. The treatment eligibility rate of 6.4% reported in this study (based on the 2015 WHO criteria) is much lower than the reported rate of 46.8% in the United States [40]. This low rate implies that a significant proportion of the patients were diagnosed at a stage of the disease that required close monitoring but did not warrant the immediate initiation of antiviral therapy. The 2015 WHO guidelines were more restrictive, primarily targeting patients with advanced liver disease (such as cirrhosis) and high viral loads. This conservative approach prioritized antiviral therapy for those at the highest risk of disease progression. The expanded criteria in the 2024 WHO guidelines [20] (released in March 2024) resulted in 93 participants (42.3%) being eligible for treatment. The updated guidelines incorporate broader indications for therapy, including a lower cut-off for the APRI score (0.5 instead of 2.0), an ALT level above the upper limit of normal instead of two times the upper limit of normal, and comorbidities such as diabetes mellitus. This reflects a shift towards more proactive management to prevent disease progression at an earlier stage, potentially reducing the incidence of liver cirrhosis and hepatocellular carcinoma. Broader treatment can also lower the overall viral load within the community, decreasing transmission rates and contributing to public health efforts to control hepatitis B. Patients eligible under the 2024 guidelines can also benefit from improved quality of life through early intervention. This includes better management of symptoms and reduced anxiety related to disease progression. Ensuring a steady supply of antiviral medications is essential to prevent interruptions in treatment.

Implementing the 2024 guidelines may require increased resources, including antiviral medications, diagnostic tools, and healthcare personnel trained in hepatitis B management. This could strain the healthcare system in resource-limited settings like Ghana but offers long-term benefits in reducing hepatitis B-related morbidity and mortality. While the initial investment in broader treatment may be high, the long-term savings from reduced healthcare costs associated with managing advanced liver disease could be substantial. Healthcare providers need updated training on the new guidelines to effectively identify and manage eligible patients. Enhancing laboratory and diagnostic infrastructure is also crucial to support the broader eligibility criteria.

To be able to accomplish the WHO goal of eliminating viral hepatitis by the year 2030, it is important to not only precisely estimate treatment eligibility rates but also to ensure high treatment rates. To the best of our knowledge based on currently available literature this is the first study that evaluates treatment eligibility (rate) among HBV infected patients seeking medical care in Ghana based on the updated WHO 2024 treatment guidelines. Evaluation of treatment rate i.e., proportion of patients eligible for treatment who receive antiviral treatment was beyond the scope of this study and remains an outstanding question for future studies. Ongoing data collection and analysis are needed to monitor the impact of the expanded guidelines and make necessary adjustments. Further research should also be conducted to assess the long-term outcomes of patients treated under the new criteria, providing evidence to support ongoing policy adjustments.

### Limitations

This study was conducted among patients who had voluntarily reported at the viral hepatitis/ STIs clinic to seek medical care for HBV infection and thus did not include in-patients admitted at the medical emergency unit or on the medical wards for complications of hepatitis B infection. Neither did the study capture HBV patients who were not seeking hospital-based care at all. Thus, the results of this study may potentially underestimate the treatment eligibility rate and the prevalence of advanced disease among HBsAg seropositive patients in the study population. Hepatitis D coinfection is now considered a criterion for the initiation of antiviral therapy in the 2024 WHO guidelines. However, routine hepatitis D testing was not conducted among the patients in this study. Thus, the treatment eligibility rate reported in this study does not include the cohort of patients who would have qualified for the initiation of antiviral therapy solely based on confirmed hepatitis D coinfection.

## Conclusions

This study reports a high level of HBsAg positivity rate among close family members of HBV positive persons. Nonetheless, there is very low adherence to the requirement of screening of close family members of index cases. There is a very low treatment eligibility rate among patients seeking medical care for HBV infection. There was low prevalence of both HIV and HCV coinfections without any recorded cases of triple infections. The study underscores the significant impact of updated WHO guidelines on hepatitis B treatment eligibility. The transition from the 2015 to the 2024 guidelines marks a progressive step towards comprehensive hepatitis B management, with the potential to improve patient outcomes and public health significantly. However, successful implementation will require concerted efforts in resource allocation, healthcare infrastructure development, and continuous monitoring.

## Supporting information

S1 File Dataset. CC: Cape Coast, BMI: Body mass index, HIV: Human immunodeficiency virus, HCV: Hepatitis C virus, HBV: Hepatitis B virus, DNA: Deoxyribonucleic acid, eGFR: Estimated glomerular filtration rate.
(RAR)

## References

[1] Razavi-Shearer D, Gamkrelidze I, Pan C, Jia J, Berg T, Gray R, et al. Global prevalence, cascade of care, and prophylaxis coverage of hepatitis B in 2022: a modelling study. Lancet Gastroenterol Hepatol. 2023;8(10):879–907. doi: 10.1016/S2468-1253(23)00197-8 37517414

[2] Ott JJ, Stevens GA, Groeger J, Wiersma ST. Global epidemiology of hepatitis B virus infection: new estimates of age-specific HBsAg seroprevalence and endemicity. Vaccine. 2012;30(12):2212–9. doi: 10.1016/j.vaccine.2011.12.116 22273662

[3] World Health Organization. Global hepatitis report 2017. Geneva: World Health Organization; 2017. https://www.who.int/publications/i/item/9789241565455, accessed 12 January 2024.

[4] World Health Organization. Hepatitis B, Key facts 2019. https://www.who.int/news-room/fact-sheets/detail/hepatitis-b, accessed 12 February 2024.

[5] Lavanchy D. Hepatitis B virus epidemiology, disease burden, treatment, and current and emerging prevention and control measures. J Viral Hepat. 2004;11(2):97–107. doi: 10.1046/j.1365-2893.2003.00487.x 14996343

[6] World Health Organization. Global Health Estimates. 2019. The importance of hepatitis B and C control and elimination. World Hepatitis Alliance; 2024. https://www.worldhepatitisalliance.org/resource/globalinvestment-case-for-hepatitis-b-and-c-elimination-full-resource/, accessed 1 February 2024.

[7] October is Liver Cancer Awareness Month. Geneva: World Hepatitis Alliance; 2023. https://www.worldhepatitisalliance.org/news/liver-cancer, accessed 1 February 2024.

[8] World Health Organization. Hepatitis B. 2023. https://www.who.int/news-room/fact-sheets/detail/hepatitis-b, accessed 1 February 2024.

[9] Blankson A, Wiredu E, Gyasi R, Adjei A, Tettey Y. Sero-prevalence of Hepatitis B and C viruses in cirrhosis of the liver in Accra, Ghana. Ghana Med J. 2005:39(4):132.

[10] Ofori-Asenso R, Agyeman AA. Hepatitis B in Ghana: a systematic review & meta-analysis of prevalence studies (1995–2015). BMC Infect Dis. 2016;16:130. 26987556 10.1186/s12879-016-1467-5 PMC4797341

[11] EASL. Clinical Practice Guidelines on the management of hepatitis B virus infection. J Hepatol. 2017:67(2):370–98. 28427875 10.1016/j.jhep.2017.03.021

[12] Terrault NA, Bzowej NH, Chang KM, Hwang JP, Jonas MM, Murad MH, et al. AASLD guidelines for treatment of chronic hepatitis B. Hepatology. 2016;63(1):261–83. doi: 10.1002/hep.28156 26566064 PMC5987259

[13] Global hepatitis report 2024: action for access in low- and middle-income countries. Geneva: World Health Organization; 2024. https://www.who.int/publications/i/item/9789241565455, accessed 1 February 2024.

[14] Saldanha J, Gerlich W, Lelie N, Dawson P, Heermann K, Heath A, et al. An international collaborative study to establish a World Health Organization international standard for hepatitis B virus DNA nucleic acid amplification techniques. Vox Sang. 2001;80(1):63–71. doi: 10.1046/j.1423-0410.2001.00003.x 11339072

[15] Wang J, Zhao S, Su Z, Liu X. Analytical comparison between two hematological analyzer systems: Mindray BC-5180 vs Sysmex XN-1000. J Clin Lab Anal. 2019;33(8):e22955. doi: 10.1002/jcla.22955 31218736 PMC6805265

[16] Branson BM, Handsfield HH, Lampe MA, Janssen RS, Taylor AW, Lyss SB, et al. Revised recommendations for HIV testing of adults, adolescents, and pregnant women in health-care settings. MMWR Recomm Rep. 2006;55(RR-14):1–17; quiz CE1-4. 16988643

[17] Smith BD, Jewett A, Drobeniuc J, Kamili S. Rapid diagnostic HCV antibody assays. Antivir Ther. 2012;17(7 Pt B):1409–13. doi: 10.3851/IMP2470 23322678 PMC5791540

[18] Wai CT, Greenson JK, Fontana RJ, Kalbfleisch JD, Marrero JA, Conjeevaram HS, et al. A simple noninvasive index can predict both significant fibrosis and cirrhosis in patients with chronic hepatitis C. Hepatology. 2003;38(2):518–26. doi: 10.1053/jhep.2003.50346 12883497

[19] World Health Organization. Guidelines for the prevention, care and treatment of persons with chronic hepatitis B infection: Mar-15. Geneva: World Health Organization; 2015 Aug 5. https://www.who.int/publications/i/item/9789241549059, accessed 12 January 2024. 26225396

[20] World Health Organization. Guidelines for the prevention, diagnosis, care and treatment for people with chronic hepatitis B infection. Geneva: World Health Organization; 2024. https://www.who.int/publications/i/item/guidelines-for-the-prevention-diagnosis-care-and-treatment-for-people-with-chronic-hepatitis-b-infection, accessed 12 June 2024. 40424433

[21] Duah A, Nartey YA. Clinical Profile and Limitations in the Management of HBV Patients Attending Clinic at a District Hospital in Ghana. Int J Hepatol. 2023;2023:4424718. doi: 10.1155/2023/4424718 36643337 PMC9833894

[22] Howell J, Lemoine M, Thursz M. Prevention of materno-foetal transmission of hepatitis B in sub-Saharan Africa: the evidence, current practice and future challenges. J Viral Hepat. 2014;21(6):381–96. doi: 10.1111/jvh.12263 24827901

[23] Antwi-Baffour SS, Adarkwah-Yiadom K, Kyeremeh R, Adjei DN, Abdulai MS, Ayeh-Kumi PF. Incidence of hepatitis B surface antigen among sickle cell disease patients receiving transfusion therapy. Int J Biomed Sci Engineering. 2014;2(1):7–10.

[24] Adjei AA, Armah HB, Gbagbo F, Ampofo WK, Boamah I, Adu-Gyamfi C, et al. Correlates of HIV, HBV, HCV and syphilis infections among prison inmates and officers in Ghana: A national multicenter study. BMC infectious diseases. 2008;8(1):1–12. 18328097 10.1186/1471-2334-8-33 PMC2311310

[25] Fenny AP, Asante FA, Arhinful DK, Kusi A, Parmar D, Williams G. Who uses outpatient healthcare services under Ghana’s health protection scheme and why? BMC Health Serv Res. 2016;16(1):174. doi: 10.1186/s12913-016-1429-z 27164825 PMC4862147

[26] Coombs RW, Reichelderfer PS, Landay AL. Recent observations on HIV type-1 infection in the genital tract of men and women. AIDS. 2003;17(4):455–80. doi: 10.1097/00002030-200303070-00001 12598766

[27] Baig S. Gender disparity in infections of Hepatitis B virus. J Coll Physicians Surg Pak. 2009;19(9):598–600. 19728952

[28] Vittal A, Ghany MG. WHO Guidelines for Prevention, Care and Treatment of Individuals Infected with HBV: A US Perspective. Clin Liver Dis. 2019;23(3):417–32. doi: 10.1016/j.cld.2019.04.008 31266617 PMC9616205

[29] Kittner JM, Brokamp F, Jäger B, Wulff W, Schwandt B, Jasinski J, et al. Disclosure behaviour and experienced reactions in patients with HIV versus chronic viral hepatitis or diabetes mellitus in Germany. AIDS Care. 2013;25(10):1259–70. doi: 10.1080/09540121.2013.764387 23383628

[30] Adjei CA, Stutterheim SE, Naab F, Ruiter RA. “To die is better than to tell”: reasons for and against disclosure of chronic hepatitis B status in Ghana. BMC public health. 2020;20(1):1–9. 32398150 10.1186/s12889-020-08811-5 PMC7216649

[31] Mahajan AP, Sayles JN, Patel VA, Remien RH, Sawires SR, Ortiz DJ, et al. Stigma in the HIV/AIDS epidemic: a review of the literature and recommendations for the way forward. AIDS. 2008;22 Suppl 2(Suppl 2):S67–79. doi: 10.1097/01.aids.0000327438.13291.62 18641472 PMC2835402

[32] Kafeero HM, Ndagire D, Ocama P, Walusansa A, Sendagire H. Sero-prevalence of human immunodeficiency virus-hepatitis B virus (HIV-HBV) co-infection among pregnant women attending antenatal care (ANC) in sub-Saharan Africa (SSA) and the associated risk factors: a systematic review and meta-analysis. Virol J. 2020;17(1):170. doi: 10.1186/s12985-020-01443-6 33160386 PMC7648981

[33] Lemoine M, Eholie S, Lacombe K. Reducing the neglected burden of viral hepatitis in Africa: strategies for a global approach. J Hepatol. 2015;62(2):469–76. doi: 10.1016/j.jhep.2014.10.008 25457207

[34] Nartey YA, Okine R, Seake-Kwawu A, Ghartey G, Asamoah YK, Senya K, et al. A nationwide cross-sectional review of in-hospital hepatitis B virus testing and disease burden estimation in Ghana, 2016–2021. BMC Public Health. 2022;22(1):2149. doi: 10.1186/s12889-022-14618-3 36419017 PMC9686031

[35] Kwasikumah F, Osisiogu EU, Asumang P, Collins A, Close A, Ringway O. Assessment of public opinion on the use of herbal medicine for the treatment of sexually transmitted infections (STI) in Accra. Trad Med Mod Med. 2024;7:1–12.

[36] Aziato L, Antwi HO. Facilitators and barriers of herbal medicine use in Accra, Ghana: an inductive exploratory study. BMC Complement Altern Med. 2016;16:142. doi: 10.1186/s12906-016-1124-y 27229306 PMC4880958

[37] Amegbor PM. Understanding usage and preference for health care therapies in a Ghanaian context: A pluralistic perspective. Nor Geogr Tidsskr. 2017;71(5):288–300.

[38] Guirgis M, Nusair F, Bu YM, Yan K, Zekry AT. Barriers faced by migrants in accessing healthcare for viral hepatitis infection. Intern Med J. 2012;42(5):491–6. doi: 10.1111/j.1445-5994.2011.02647.x 22151101

[39] Giles-Vernick T, Hejoaka F, Sanou A, Shimakawa Y, Bamba I, Traore A. Barriers to Linkage to Care for Hepatitis B Virus Infection: A Qualitative Analysis in Burkina Faso, West Africa. Am J Trop Med Hyg. 2016;95(6):1368–75. doi: 10.4269/ajtmh.16-0398 27928086 PMC5154452

[40] Wong RJ, Jain MK, Therapondos G, Niu B, Kshirsagar O, Thamer M. Low rates of hepatitis B virus treatment among treatment-eligible patients in safety-net health systems. J Clin Gastroenterol. 2022;56(4):360–8. doi: 10.1097/MCG.0000000000001530 33780210

